# Metal nanoparticle alters adenine induced charge transfer kinetics of vitamin K3 in magnetic field

**DOI:** 10.1038/s41598-020-75262-8

**Published:** 2020-10-28

**Authors:** Ranjan Kumar Behera, Abhishek Sau, Leepsa Mishra, Sankalan Mondal, Kallol Bera, Satish Kumar, Samita Basu, Manas Kumar Sarangi

**Affiliations:** 1 Department of Physics, Indian Institute of Technology Patna, Patna, India; 2 Chemical Sciences Division, Saha Institute of Nuclear Physics, Kolkata, India; 3 Present Address: Department of Molecular and Cellular Medicine, Texas A&M University, College Station, USA; 4 Present Address: Division of Biology and Biological Engineering, California Institute of Technology, Pasadena, USA

**Keywords:** Materials science, Nanoscience and technology

## Abstract

In this article, we highlight the alterations in the photoinduced electron transfer (ET) and hydrogen atom transfer (HAT) pathways between an anti-tumor drug vitamin-K3 (MQ) and a nucleobase adenine (ADN) in the presence of gold (Au) and iron (Fe) nanoparticles (NPs). Inside the confined micellar media, with laser flash photolysis corroborated with an external magnetic field (MF), we have detected the transient geminate radicals of MQ and ADN, photo-generated through ET and HAT. We observe that the presence of AuNP on the MQ-ADN complex (^Au^MQ-ADN) assists HAT by limiting the ET channel, on the other hand, FeNP on the MQ-ADN complex (^Fe^MQ-ADN) mostly favors a facile PET. We hypothesize that through selective interactions of the ADN molecules with AuNP and MQ molecules with FeNP, a preferential HAT and PET process is eased. The enhanced HAT and PET have been confirmed by the escape yields of radical intermediates by time-resolved transient absorption spectroscopy in the presence of MF.

## Introduction

Dictated by several quantum mechanical effects, metal nanoparticles, compared to their bulk, possess many unique physical and chemical properties. Owing to the nano-confinement, novel characteristics like surface plasmons, size-dependent optoelectronic behavior, surface functionality, etc. have invited applications in fields of imaging, catalysis, medicine, sensing, electronics etc^1–6^. Out of the many different metal NPs, AuNP, AgNP, and FeNPs have observed an upsurge for their size compatible engineering and unique nanoscale physiochemical properties^7–9^. Versatile surface modifications and functionalization of the NP, such as PEGylation^10,11^, crosslinking^12^, encapsulation^13^, grafting^14^, coating^15,16^, etc. often lead to additional functionality like higher colloidal stability and biocompatibility, multimodal imaging, biosensing, and therapy with target specificity. Iron oxide nanoparticles (FeNP) have been used for magnetic force guided targeted drug delivery, magnetic resonance imaging (MRI) contrast agent for sensitive and accurate diagnosis, navigation, sensing, imaging, therapy, magneto-thermal and magneto-mechanical treatment^17,18^. On the other hand, plasmonic nanoparticles like AuNP have low cell toxicity and have the advantage of having biocompatible surface passivation. This enables AuNP to get easily tagged to biomolecules, and hence act as real-time reporters to unveil complicated biomolecular reactions and also mediate photocatalytic activity with higher efficacy^19–21^. Due to the abundance of electrons at their surfaces, they take part in charge transfer reaction with nearby molecules through photoinduced electron transfer (PET), hydrogen atom transfer (HAT) along with radiative and nonradiative energy transfer^22–27^, etc.

How these NPs interfere and modulate the regular catalytic activities of the native biomolecules inside the cell often decide their efficacy, toxicity, and usage as nanomedicine^3,28^. Cell inherently possesses many natural photo-active molecules, which are responsible for the several catalytic reactions for its survival. The redox reactions often involve electron or hydrogen atom transfer in a sequential or concerted way to generate highly reactive radical ion intermediates with the unusually low activation barrier for efficient catalytic photochemical transformation^29,30^. Nature’s ability to carry out such photo-redox reactions with ease has inspired various research groups to develop photocatalysts for mimicking these reactions with notable applications in water splitting, solar energy storage, photovoltaics etc^31,32^. Quinones are one of such ubiquitous compounds found abundantly in several natural products and serve as an active electron-proton shuttle in a variety of processes involving intra or intercellular charge transport^33–35^. In photosynthesis and respiration chain, quinones play a pertinent role in electron and water-assisted proton-coupled electron transfer phenomena forming active radicals. Here, we have studied a bioactive quinone analog vitamin K3 [menadione (MQ)], known for its redox-modulating properties, particularly its role in calcium homeostasis^36–38^, prevention of mitochondrial dysfunction^39,40^ and onco-suppressive effect^41–45^. The anti-tumor activities are often ascribed to the redox-cycling and the synergy to produce endogenous reactive oxygen species^46^. MQ forms semiquinones and hydroquinone radicals by one and two-electron reductions, respectively, accompanied by the consumption of superoxide radicals. An increase in superoxide in malignant cells induces apoptosis and cell death^46^.

In this report, we address the alteration in the normal photophysical behavior of MQ with adenine (ADN) in the presence of two types of NPs, e.g., AuNP and FeNP. Sengupta et al. have reported that the photoinduced charge transfer interaction between adenosine and MQ involves both PET and HAT, and their rates are constrained by the polarity of the intervening surrounding media^47^. Photoinduced electron transfer from donor to acceptor often create geminate radical pairs (RPs) and radical ion pairs (RIPs) containing free electrons, which are susceptible to applications of an external magnetic field (MF). The spin-correlated geminate RPs/RIPs undergo spin flipping or rephasing, with a suitable MF of hyperfine order. With an optimal separation distance, where exchange interactions become negligible, the intersystem crossing (ISC) between the singlet (S) and triplet (T) of the RIPs/RPs is maximized^48^. With the application of MF, Zeeman splitting lifts the degeneracy of the T states and hence reduces the ISC between them. This results in an increase in the populations of initial spin states of the RIPs^49^. The criticality of the optimum distance is maintained by controlling the dielectric of the medium or by encapsulating the radicals in organized assemblies like micelles, reverse micelles, and vesicles. This also helps in preventing the back electron transfer and hence prolong the lifetime of the formed radicals^49,50^.

By virtue of their composition, biological macromolecules provide confined and restricted environments required to sustain the geminate characteristics of the RIPs^51^. However, the distance-dependent MF has also been observed in a linked system comprised of varied inter-radical distance^52,53^. The studies of magnetic field effect (MFE) with nanomaterials have been reported earlier by E. Cohen^54^. Das Chakroborty et al. showed the occurrence of H-abstraction in the presence of AuNP and FeNP with sanguinarine, which acts as a toxin that kills animal cells through its action on the Na^+^/K^+^-ATPase transmembrane protein^55,56^. A highly directional MF can be generated by a single domain superparamagnetic Fe_3_O_4_ nano surface in the presence of a low external MF (0.08 T) on the nano surface adsorbed molecules. The magnetic nano surface can enforce the transient spin-trajectory through S_1_ → T_1_ interconversion of surface adsorbed sanguinarine molecule, through the enhanced population of the spin-rephased component in the triplet nondegenerate state. The superparamagnetic moment helps to achieve a favourable orientation in the same direction instead of the random angle in the presence of an external low MF (0.08 T) to assist the projected spin interconversion. Moreover, the polarity and the protic nature of the solvent molecules control the transient spin trajectory distribution. Herein, we report whether the NPs, which preferentially adsorb MQ and ADN, can alter their excited state reaction pathways and their dynamics in an external MF, by detecting transient intermediates using laser flash photolysis.

## Experimental methods

All the solvents and chemicals used for the synthesis of gold and iron nanoparticles are of analytical grade. The reagents ADN, MQ, sodium dodecyl sulfate (SDS), HAuCl_4_, tri-sodium citrate (TSC), and FeCl_3_, FeSO_4_, NH_4_OH, NaOH, and HCl are purchased from Sigma Aldrich. MilliQ water is used as a solvent. We have prepared all the samples in 5% SDS well above the critical micellar concentrations.

### Synthetic protocol

#### The synthetic protocol of gold nanoparticle (AuNP)

Spherical Au nanoparticles (AuNP) are synthesized by the TSC-based method reported earlier^55^. Briefly, a three-necked round bottom flask bottle fitted with a cold-water jacket condenser is filled with 100 ml of water in which 1.25 ml of 10^−2^ M aqueous solution of HAuCl_4_ is added to it. Then it is heated to boil, and 0.75 ml of 1% of TSC (0.022 mM) is added at a time under that condition. The heating is continued up to the next 30 min to complete the reaction. Eventually, the reaction is stopped, and the reactants are cooled to room temperature under stirring conditions. The synthesized AuNP stock solution is centrifuged at 6500 rpm speed for 1 h, and the precipitation is washed out by MilliQ water several times to get AuNP.

#### The synthetic protocol of iron nanoparticle (FeNP)

The magnetic iron nanoparticle (FeNP) is synthesized by the chemical co-precipitation method by reducing FeSO_4_ and FeCl_3_ ions (1:2 molar ratio) in the presence of NH_4_OH as reported by Das Chakraborty et al.^56^. In the process of preparation, a three-necked flask containing 50 ml of water solution of 0.01 mol/L FeSO_4_ and 0.02 mol/L FeCl_3_ (25 ml each) are taken. The temperature of the flask is raised to 80 °C to keep the solution in reflux condition under the argon atmosphere, and then it is stirred vigorously at 1200 rpm speed. A 10 ml solution of NH_4_OH (13.4 M) is added instantly whenever the temperature reaches 80 °C, which increases the pH of the solution to 11.0. Then TSC (0.5 g/ml) is added, and the mixture is stirred for 60 min at 90 °C. The black precipitation is collected after proper cooling. The suspension is washed several times with deionized water to remove excess citrate ions.

#### Preparations of the samples

The complexes of MQ-ADN are prepared in-situ taking the molar ratio of MQ (0.1 mM) and ADN (2 mM) in 5% SDS micelles. The amount of ADN is taken in excess to ensure all MQ are in the complex. The complex is then vortexed to get a homogeneous transparent solution. ^Au^MQ-ADN: After the preparation of MQ-ADN, we have added the desired concentration of AuNP (2.8 μg/ml) to the MQ-ADN, and the mixture is then vortexed to form a uniform solution of ^Au^MQ-ADN. ^Fe^MQ-ADN: Similar procedure is also followed by the desired concentration of FeNP (2.8 μg/ml) with MQ-ADN for the preparation of ^Fe^MQ-ADN. Throughout the experiments, the pH of the solutions is maintained at 7.4.

### Steady-state measurement

The steady-state absorption measurement is carried out by JASCO V-650 UV–Visible absorption spectrophotometer with 10 mm path length quartz cuvettes. All measurements are done with proper baseline correction and inner filter effect correction at room temperature.

### Laser flash photolysis

The triplet transient (T–T) intermediate species are recorded with third harmonics output of nanosecond flash photolysis setup (applied photophysics) containing Nd: YAG (Lab series, model lab 150, Spectra-Physics) where a pulsed xenon lamp (150 W) is used to monitor the species. The laser wavelength 355 nm with fwhm = 8 ns is used to excite the sample. A Tektronix oscilloscope (TDS 3054B, 500 MHz,5 Gs/s), is connected to the output of the photomultiplier (1P28), and the average data of 3 shots are transferred to a computer using a TekVISA software. More details of the laser flash photolysis setup are explained elsewhere^57,58^. The effect of the MF on the transient species are observed by supplying a direct current through a pair of electromagnets (having field strength 0.08 T) placed inside the sample chamber. The sample is taken in a laser cuvette with a path length 10 mm and is degassed by passing through argon gas for 20 min before each experiment. The pH of all the reaction mixtures is adjusted to 7.4 by adding an appropriate amount of NaOH and/or HCl. For data plotting and fitting of the decay curve, origin 8.5 software is used in the B-spline option. The average lifetimes, radical escape yields, and lifetime yields are obtained by the following relation.

1 $$ Average\,life\,time\, \left\langle \tau \right\rangle = \frac{{A_{1} \tau_{1}^{2} + A_{2} \tau_{2}^{2} }}{{A_{1} \tau_{1} + A_{2} \tau_{2 } }} $$

$$ \begin{aligned} Radical\,escape\,yield\,\left( {REY} \right) & = \frac{{O.D. _{MF } }}{{O.D._{ WMF} }} \\ life\,time\,yield\,\left( {LY} \right) & = \left( {\frac{{\tau _{MF } }}{{\tau_{WMF} }} - 1 } \right) \times 100\% \\ \end{aligned} $$

We have analyzed the decay profiles (change in absorbance A(t) versus time t at a particular wavelength λ) as shown in Figure S2,S3 and S4, in the absence and presence of MF following the equation:

2 $$ A\left( t \right) = B_{1} \exp \left( { - k_{f} t} \right) + B_{2} \exp \left( { - k_{s} t} \right);\quad k_{f} = \frac{1}{{\tau_{f} }}\quad and\quad k_{s} = \frac{1}{{\tau_{s} }} $$

*B*_1_ and *B*_2_ are the amplitude of the transients, where *k*_*f*_ and *k*_*s*_ are the respective rate constants for the fast and slow components of the corresponding decay profile^59^.

### Transmission electron micrograph

Transmission electron microscopic measurements are recorded by a FEI, Tecnai G2 F30, S-Twin microscope operating at 300 kV equipped with a GATAN Orius SC1000B CCD camera. We have used a simple drop-cast technique to prepare the sample. A 300-mesh carbon-coated copper grid followed by drop-cast of the required sample is taken and kept it in vacuum condition for 24 h to evaporate the solvent. After complete drying, the grid is used for measurement, and the images are analyzed through ImageJ software.

### Deconvolution of transient absorption spectra

The transient absorption spectra are first fitted with the summation of three Gaussian functions with a peak value at 380 nm, 410 nm, and 520 nm. Python 3.7 is used for curve_fitting with the option of optimizing function, which belongs to SciPy library. The curve_fit uses the Levenberg–Marquardt algorithm for the least square optimum fit to find the fitted parameters. The optimized function provides amplitude and sigma for each Gaussian function, which is later used to deconvolute the contribution of the respective Gaussian function. To calculate the contribution of each Gaussian function, their area under the curve is normalized.

## Results and discussions

**Figure 1:**
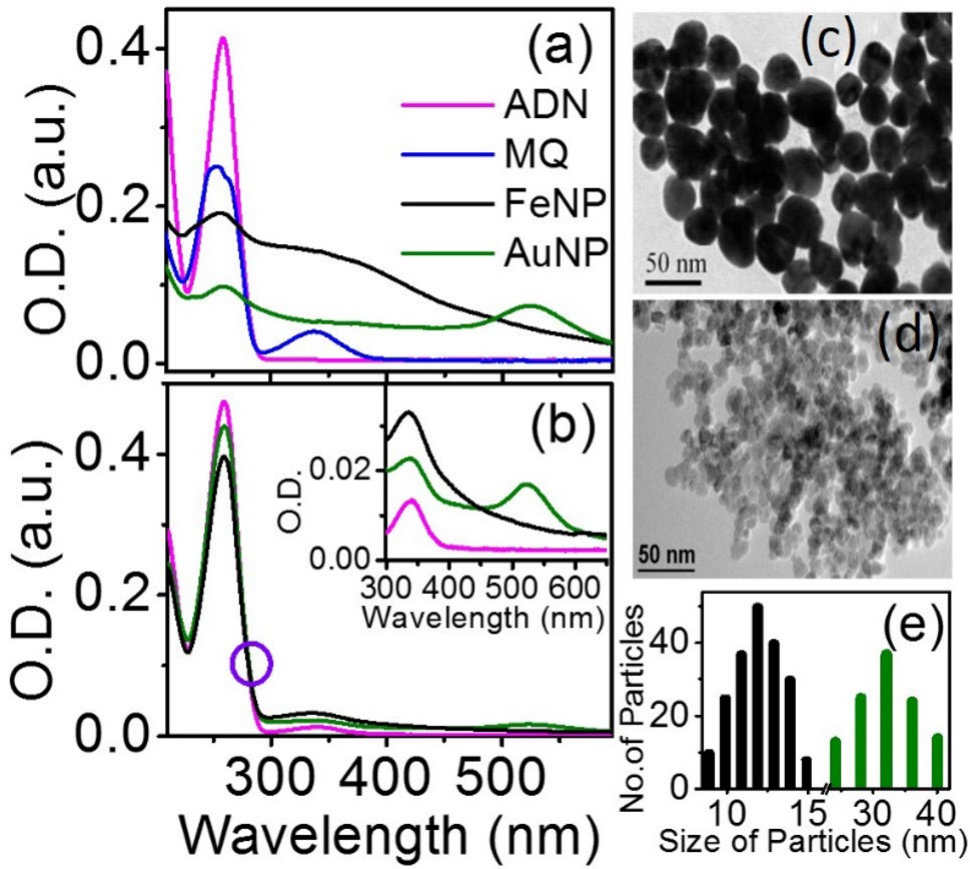
Steady state absorption spectra of (**a**) MQ (0.1 mM, blue), ADN (2 mM, magenta), AuNP (2.8 μg/ml, olive), FeNP (2.8 μg/ml, black) in 5% SDS (**b**) MQ-ADN complex (magenta), ^Au^MQ-ADN (olive) and ^Fe^MQ-ADN (black) in 5% SDS, the inset shows the zoomed view of figure (**b**) from 300 to 600 nm wavelength region. High resolution transmission electron microscopy (HRTEM) image of (**c**) AuNP and (**d**) FeNP and (**e**) their histogram (FeNP-black, AuNP-Olive) over diameter and number of particles.

A characteristic absorption spectrum of MQ has two peaks around 260 and 340 nm corresponding to π–π* and n–π* transition, while the ADN has one sharp absorption peak at 260 nm (Fig. 1a)*.* The UV–visible absorption of 2.8 μg/ml of AuNP (olive line) and FeNP (black line) have been shown in Fig. 1a with the characteristic surface plasmon band for AuNP and a gradually decreasing absorption for the FeNP as reported in the literature^55,56^. The addition of AuNP or FeNP to the complex MQ-ADN shows a decrease in the 260 nm peak with a concomitant increase in the peaks over the red end, with an isosbestic point at 280 nm (Fig. 1b). This indicates that the nanoparticles are perturbing the interactions of ADN and MQ in the micellar assemblies. The inset of Fig. 1b shows a zoomed view of the different absorption bands of the NPs and MQ in the range 300–600 nm. The HRTEM images of AuNP and FeNP and their size distributions are shown in Fig. 1c–e, respectively. The prepared NPs have an average diameter of around 30 nm and 12 nm for the AuNP and FeNP, respectively. Though FeNP, ADN, and MQ have very strong UV absorption, they possess a very short singlet lifetime; however, reported literature states that they show strong triplet–triplet (T–T) absorption bands^47,60^. Previous works have indicated quinones have faster intersystem crossing (k_ISC_) compared to k_ET_ with higher triplet quantum yields and short residence time at the excited singlet state, and hence the photoinduced electron transfer processes often occur in the triplet state of the quinone and can be readily detected^61–64^. This helps in easy detections of the radical products with longer triplet state lifetimes and higher transient absorption. Moreover, as reported by Amada et al., and also by Sengupta et al., the HAT processes occur in the triplet state of the menadione molecule^47,65–67^. Due to this, it has higher T–T absorption and the respective kinetics become readily detectable.

**Figure 2:**
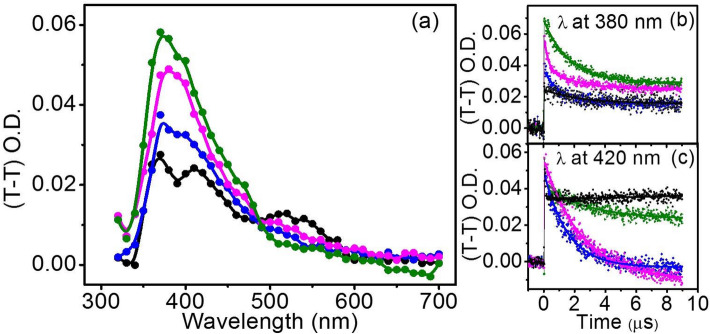
(**a**) Transient absorption spectra generated after 1 μs of laser flash (λ_ex_ = 355 nm), for (blue circle) MQ, (pink circle) MQ-ADN (green circle) ^Au^MQ-ADN, and (black circle) ^Fe^MQ-ADN in 5% SDS. Their corresponding decay profiles are shown in (**b**) 380 nm and (**c**) 420 nm wavelengths. All the experiments are performed by keeping concentrations of MQ, ADN, AuNP, and FeNP at 0.1 mM, 2 mM, 2.8 μg/ml, and 2.8 μg/ml, respectively. Similar spectra at 4 μs time delay is shown in S1.

Herein, we employ (T–T) transient absorption spectroscopy for deciphering their underlying photoinduced interaction pathways between ADN and MQ under the influence of NPs. The transient absorption spectra of argon-saturated solutions of MQ and their mixtures MQ-ADN, ^Au^MQ-ADN, ^Fe^MQ-ADN in SDS are obtained by irradiation of the samples separately by a 355 nm laser pulse (Fig. 2). The T–T absorption of MQ only shows two major convoluted peaks at 380 nm and 420 nm with a small absorption around 520 nm at the tail (Fig. 2a). With the addition of ADN, we observe an overall rise in the two major peaks along with a small increment around 520 nm. It is reported that on photoexcitation, MQ can produce radical products by both ET and HAT depending on the surrounding media, with characteristic signatures of MQH^·^ (at 420 nm) and MQ^·−^ (at 380 and 520 nm)^47,68^. Sengupta et al. have reported that in acetonitrile media ET yield of MQ is higher, whereas the water-assisted HAT products predominate in the SDS micellar medium^47^. Here, we monitor some uncommon changes in the charge transfer radical yields at their respective wavelengths when the MQ-ADN interaction is perturbed by the presence of AuNP and FeNP. With AuNP, we have observed an enhancement in the absorbance around 380 nm with a drop in the 520 nm band. However, with the addition of FeNP, the peak around 380 nm decreases with the increase of that around 520 nm. The decrease in absorbance around 520 nm with AuNP signifies the dearth of formation of ET product MQ^·−^. Figure 2b, c represent the decay profiles of the transient radicals formed at 380 and 420 nm, respectively, with fitted lifetimes by using Eq. 1 (Table 1). While the decay profiles at 380 and 420 nm for the MQ only sample are fitted to a single exponential with a reduced chi-square, the decay profiles of MQ with ADN in the presence and absence of the NPs require biexponential for best fit by using Eq. (2). The biexponential behavior is an indication of the formation of the additional reaction product, resulting from the bimolecular interaction between MQ and ADN after photoexcitation. Both MQ^·−^ and MQH^·^ show higher absorption and an enhancement in their lifetimes from 1.12–1.75 μs to 1.5–2.2 μs, respectively. The signature of MQ^·−^ at 520 nm also reflects an increase in the lifetime from 1.32 to 1.83 μs signifying that the reaction products are stable and driven by light absorption. In the presence of both AuNP and FeNP, the decay profiles at 420 nm show distinct changes (Fig. 2c) with a very long lifetime indicating the formation of a stable complex with NPs. In the presence of FeNP, we have noticed a distinct signature of the MQ^·−^, MQH^·^ at 380 and 420 nm along with a significant increase in the absorption around 520 nm. A pronounced absorption at 380 and 520 nm implies a facilitated ET in the presence of FeNP. For AuNP, the O. D. of 520 nm peak suffers a slight drop along with an increment in the 420 nm peak, reflecting a reduction in the radicals of the ET product. Moreover, the peaks for 380 and 420 nm become more convoluted and indistinguishable, indicating the dominance of HAT radical products. These changes are accompanied by an isosbestic point around 485 nm, which reflects a dynamic equilibrium between ET and HAT products in the presence of the nanoparticles. This is in congruence with the changes in the lifetimes and the corresponding radical escape yield. The observations indicate that ET between MQ and ADN in SDS dominates over HAT in the presence of FeNP, whereas HAT radical product formation becomes more favorable on the addition of AuNP.

**Table 1:** The lifetime of transient species (τ) obtained at their respective wavelengths has been calculated in the presence and absence of a 0.08 T MF.

Magnetic field (T)	MQ		MQ-ADN		^Au^MQ-ADN		^Fe^MQ-ADN	
	0	0.08	0	0.08	0	0.08	0	0.08
*380 nm*								
τ_1_ (μs)	1.12	1.81	1.75	2.07	2.11	2.46	1.42	2.14
B_1_ (%)	–	70	38	61	74	71	35	47
τ_2_ (μs)	–	0.40	0.20	0.07	0.40	0.40	0.38	0.32
B_2_ (%)	–	30	62	39	26	29	65	53
< τ > (μs)	1.12	1.68	1.50	2.02	2.00	2.33	1.07	1.87
*420 nm*								
τ_1_ (μs)	1.50	2.06	2.20	2.56	3.10	5.75	3.09	2.15
B_1_ (%)	–	72	55	76	79	20	–	51
τ_2_ (μs)	–	0.54	0.08	0.2	0.20	0.15	–	1.79
B_2_ (%)		28	45	24	21	80	–	49
< τ > (μs)	1.50	1.91	2.13	2.50	3.05	5.22	3.09	1.99
*520 nm*								
τ_1_ (μs)	1.33	2.56	1.83	3.14	–	–	2.77	2.83
B_1_ (%)	–	28	18	20	–	–	35	94
τ_2_ (μs)	–	0.58	0.30	0.70	–	–	1.43	1.08
B_2_ (%)		72	82	80	–	–	65	06
< τ > (μs)	1.33	1.83	1.17	1.98	–	–	2.11	2.78

The parenthesis represents the amplitude of the transients obtained from the fitting (Eq. 2). The experimental error in the above measurements is less than 10%.

**Scheme 1:**
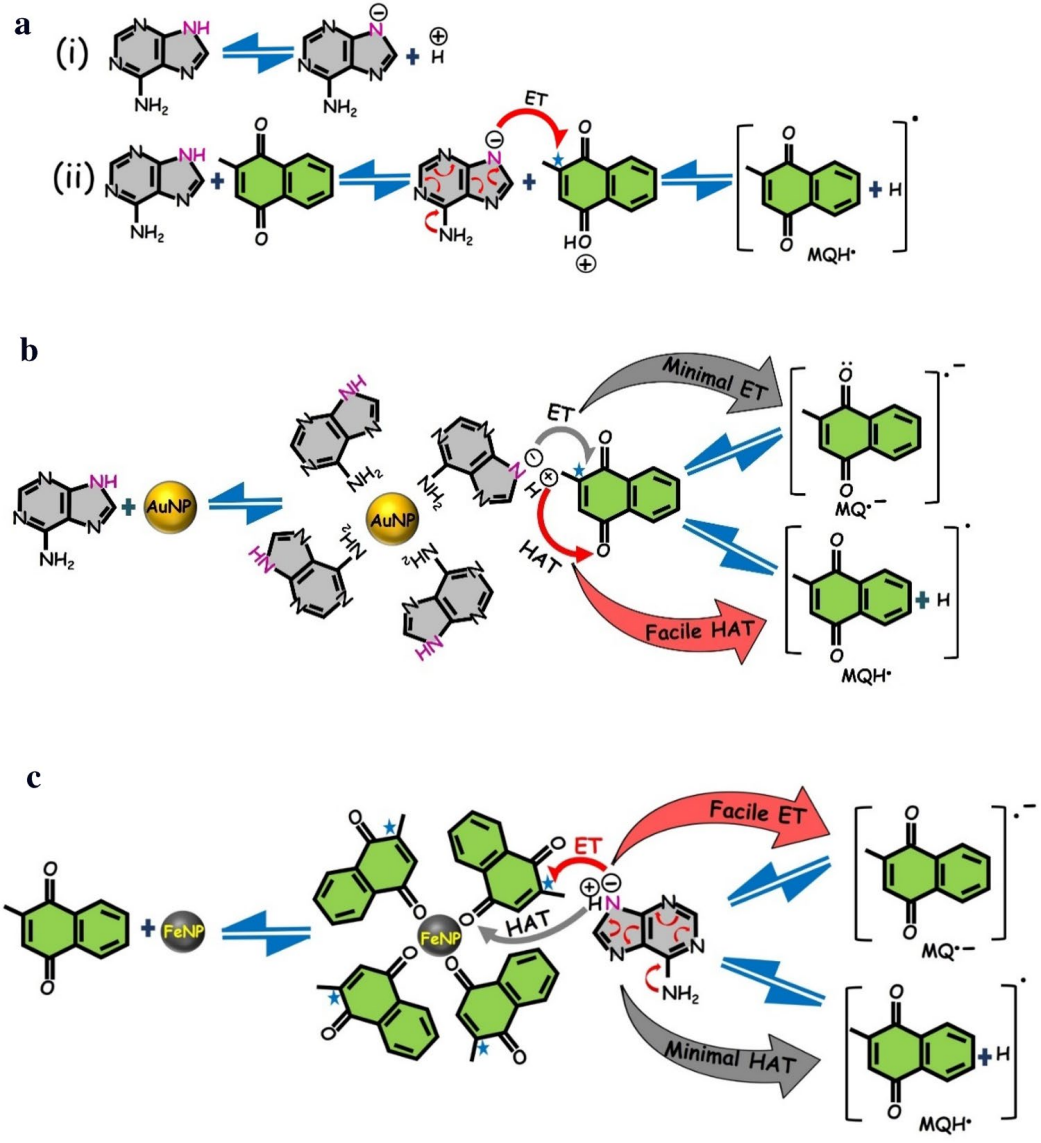
(**a**) Acid–base equilibria of ADN (i), Charge transfer (HAT and ET) between ADN and MQ. (**b**) Charge transfer (HAT and ET) between ADN and MQ in the presence of AuNP (^Au^MQ-ADN) in SDS. (**c**) Charge transfer (HAT and ET) between ADN and MQ in the presence of FeNP (^Fe^MQ-ADN) in SDS. (For the plausible structures of radical (MQH^·^) and radical anion product (MQ^·−^) of unsymmetric MQ, see Supporting Scheme 2).

ADN and its derivatives like NADP and NADPH carry out many redox reactions as a catalyst in critical cellular activities. Despite having a solid UV absorption band at 260 nm, ADN is quite stable, and its excited state lifetime is in order of few femtoseconds^69^. This is due to the opening of many swift photoinduced charge transfer pathways in the excited state of ADN, resulting in a rapid decay to the ground state through the conical intersection^69–71^. The estimation of proton release of ADN in the gas phase shows two acidic sites, N9 and N10, having energy 333 and 352 kcal mol^−1^, respectively (Supporting scheme 1)^72,73^. Therefore, the H in the 9th position of ADN will be accessed by bases much more compared to that in the 10th position (Scheme 1a–i). The lone pair of nitrogen in the -NH_2_ group does not actively participate in the resonance and hence is readily available at that position. It is well known that MQ takes part in HAT with SDS, however, when MQ is treated with ADN, the rate of HAT between ADN and MQ predominates over with SDS as the equilibrium stabilized by an associated ET from the lone pair of -NH_2_ (Scheme 1a(ii)). This is reflected in a concomitant enhancement in the transient absorbance of HAT radical around 420 nm as shown in Fig. 2. As is evident from the T–T absorption, this dynamic HAT equilibrium, and the subsequent stabilization through ET by resonance in the purine ring, is severely disrupted in the presence of the NPs. The above spectra in Fig. 2a confirm that the rate of ET is much more favored with FeNP while HAT predominates in the presence of AuNP. We hypothesize that the preferential behavior of AuNP and FeNP, particularly the yield of the HAT and radical products (Scheme 1b, c), is probably through the preferential adsorption of ADN and MQ molecules to the NPs respectively. Compared to MQ, ADN shows a higher affinity to the AuNP, due to the stronger interaction of sp^3^ N-atom of ADN compared to sp^2^ O-atom of MQ^74–78^. Now, since the amino group of ADN molecule is involved in the adsorption, the lone pair availability and hence the electron-donating ability of the amino group to the ADN N9 will decrease, which may reduce the ET to the MQ molecule. Further, as evident from Scheme 1a(i), any factor which stabilizes the conjugate ADN anion formed after proton removal will increase the release of H^+^. On binding to AuNP, since the negative charge on 9th position of ADN is used for resonance to restore the depleted electron density in the amino group and the N7 position, the H^+^ release and hence HAT is facilitated here. However, in the case of FeNP, probably a hard-hard interaction between the oxygen atom and the FeNP, MQ preferentially binds to FeNP. Also recently, it has been observed differential adsorption in NPs may result in differential reactivity^79^. A similar effect may be observed here. As MQ interacts preferentially with FeNP, ADN molecules are left free, and since the amino group of the ADN molecule is a strong electron releasing group which thereby can increase the electron density at the N9 position (through resonance) of the ADN molecule, the acidity of the N–H group at the N9 position is decreased^80,81^, and hence the HAT ability is ceased with ^Fe^MQ-ADN. Further, the amino group can also increase the electron density at the N9 position through aforesaid resonance and thus increases the ET to the MQ molecule.

**Figure 3:**
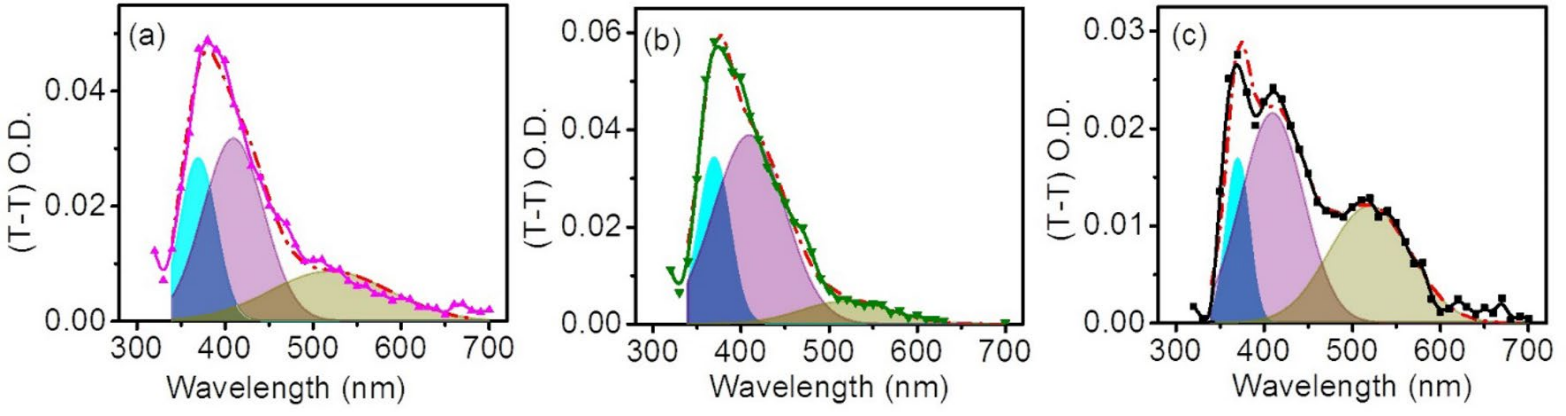
The de-convolution graph of the transient absorption spectra of (**a**) MQ-ADN (magenta), (**b**) ^Au^MQ-ADN (olive) and (**c**) ^Fe^MQ-ADN (black) respectively. Each spectrum is convoluted into three individual Gaussian functions in cyan, purple, and dark yellow color. All red lines are the fitted line to the corresponding absorption spectra at 0.5 μs time delay.

As both the ET and HAT radical products absorption peaks are adjacent to each other and overlapping in nature, the peaks become relatively indistinguishable and require a deconvolution to specifically identify the origin of the real species. We have employed a deconvolution process to monitor the extent of overlapping of the various radicals, by separately fitting to three Gaussian peak maxima at 380 nm (blue), 410 nm (pink), and 520 nm (green), respectively. The details of the deconvolution for each complex, their relative amplitude, and contribution to the integrated area is provided in Fig. 3a–c, and Table ST1. A deconvolution of the individual spectra gives the impression that, in all cases, the blue profile is narrow and is vanishing before 410 nm; however, the pink profile is broad and may have a contribution from the blue profile till 410 nm. Hence, the contribution of the pink profile after 410 nm can be safely assigned to the HAT product MQH^·^. For ET products MQ^·−^, among the two assigned peaks at 380 and 520 nm, it will be appropriate to count on the 520 nm green profile than the blue one, as it is more than 100 nm red-shifted from MQH^·^ with minimal or no contribution from it.

For MQ-ADN complex, integrated area calculation for HAT and ET infers of almost equal contributions, indicating the concurrence of both the processes maintaining the dynamic acid–base equilibria. The simultaneous occurrence of HAT and ET is abundant in many biochemical reactions, with one followed by the other, or both happening in a concerted elementary step. Quinones like MQ has been reported to readily undergo such redox processes. However, when specific NPs come into the periphery of their complex, one of the processes is favored forming stable radical products, as is evident from the increased lifetimes of the complexes in the presence of NPs. It is noteworthy to mention that, though one of the processes is facilitated, the other pathway may not be blocked entirely. Integrated area calculation suggests that in the presence of AuNP, HAT product radicals increased from ~ 49% to ~ 65%, with a concurrent drop in the ET product radicals. On the other hand, ET radical products peak ~ 520 nm shows an increment in the integrated area from ~ 26% to 36% with the addition of FeNP (Table ST1. These changes are well supported by the respective changes in the average lifetimes of MQ^·−^ and MQH^·^ with an overall change in their transient absorption peaks in the respective wavelengths. The increase in the 380 nm peak in the presence of AuNP can be attributed to the overall increment in the nearby peak at 420 nm due to HAT. According to Scaiano et al., a molecule in the proximity of metal NPs interact like a transmitter or receiver antenna in which a molecule in the ground and/or triplet excited states are anticipated with localized plasmon field in the vicinity of NP^24^. On the other hand, surface plasmon of AuNP controls the dynamics of the triplet-state of the excited molecule at 420 nm wavelength region precisely. It can be inferred that MQ produces excess MQH^·^ through the interaction with ADN adsorbed on the AuNP surface, which will include the facile HAT (Scheme 1b). Hence the overall transient absorption from 380 to 420 nm increases.

**Figure 4:**
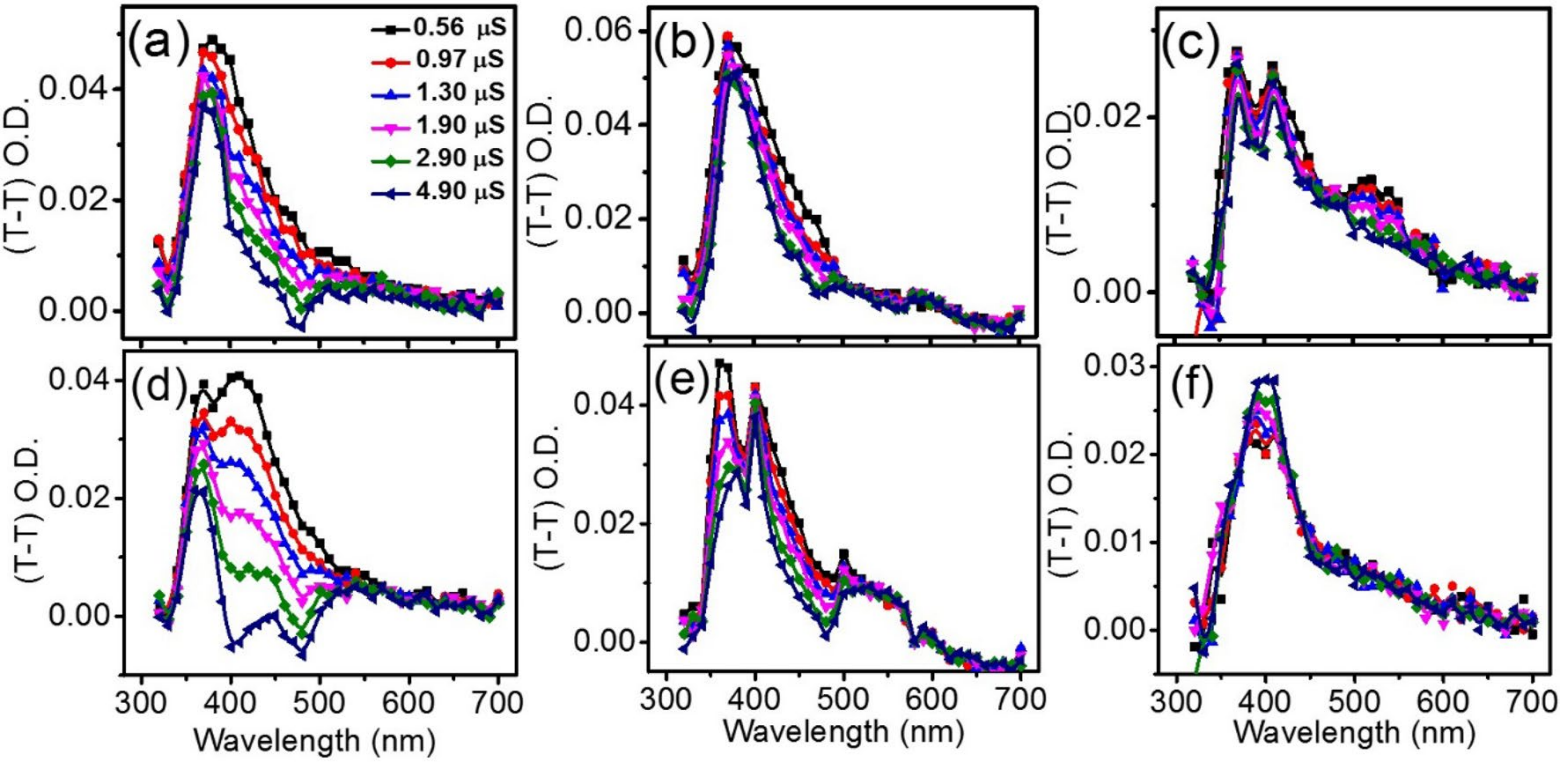
Transient absorption spectra of (**a**) MQ-ADN, (**b**) ^Au^MQ-ADN and, (**c**) ^Fe^MQ-ADN respectively generated between the pump and probe pulse delay from 0.5 to 4.9 μs time delay after the laser flash in the absence of MF and their respective spectrum in the presence of MF is shown in (**d**)–(**f**).

The transient spectrum of MQ-ADN in the presence of FeNP shows reduced peak intensity at 380 nm and 420 nm and enhanced absorbance in the region of 510–530 nm (Fig. 2). This is due to the facile ET compared to HAT through the preferential adsorption of MQ. Now, the preferential binding of the MQ with the FeNP through the lone pair on the O atom may also influence the interaction of MQ with ADN and hence the radical pair [MQ(+H)^·^ ADN(−H)^·^] formation through HAT between MQ and ADN gets reduced. Conversely, the ET is not affected and becomes prominent, as observed from Fig. 2, where the absorbance around 510 nm (MQ^·−^) increases along with a decrease in absorbance around 420 nm (MQH^·^). The absorbance around 380 nm becomes much more distinct due to the formation of counter radical cation ADN^·+^. Hence, in the presence of FeNP, ET is promoted over HAT and produces a number of ADN^·+^ and MQ^·−^. The transients formed either through HAT or ET, are further confirmed from time-resolved transient absorption spectra over the time range between 0.5 and 4.9 μs (Fig. 4a–c). The increase in absorbance around the signature wavelengths (Table 1) proves that the corresponding transients originate from HAT or ET between MQ and ADN and not from noise or fluctuations. In the absence of the NPs, we observe an overall decrease in the absorption of the radicals with longer delay time; however, in the presence of the NPs, the drop is not very significant, indicating the formation of long-lived radicals. Moreover, in the presence of AuNP, the amplitude of the MQH^·^ at 420 increases significantly, while prominent peaks for MQ^·−^ around 380 and 520 nm for FeNP remain stable even after a time delay of more than 4.9 μs.

### Magnetic field effect

**Figure 5:**
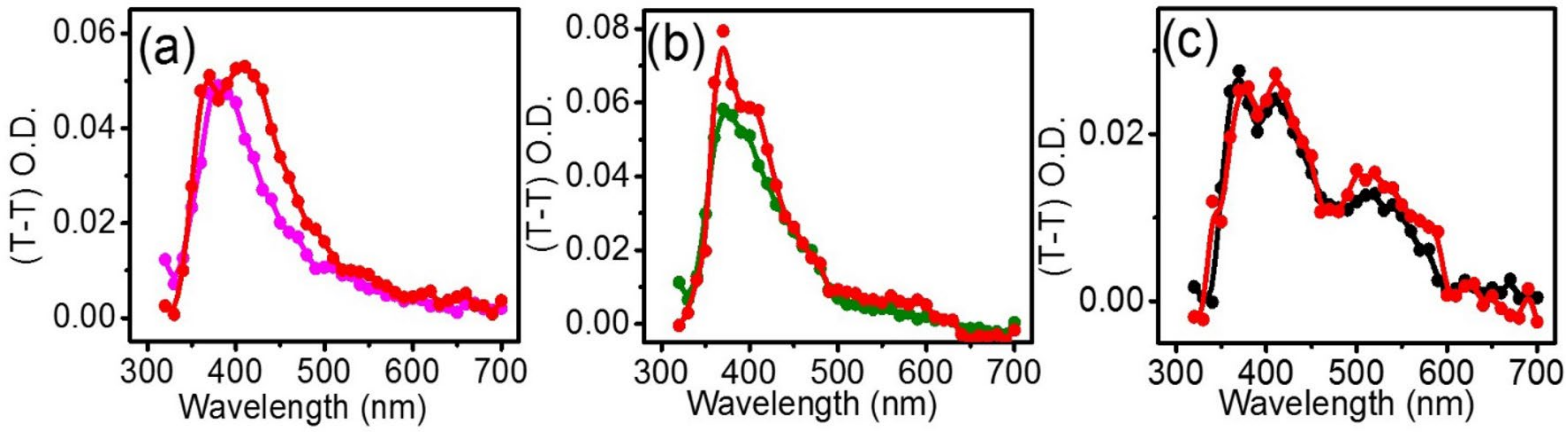
Transient absorption spectra of (**a**) ADN, (**b**) ^Au^MQ-ADN and, (**c**) ^Fe^MQ-ADN, respectively, between the pump and probe pulse at 1 μs time delay and all the red spectra represent the presence of the magnetic field for their corresponding spectra. The decay spectra at 380, 420, and 520 nm wavelength are given in supplementary information (S3).

The effect of MF on spin correlated RIPs is driven by the interplay of their spin states and diffusion dynamics. Both ET and HAT between MQ and ADN will lead to the formation of RIPs or RPs which contain unpaired electrons and hence can be affected by the application of an external MF^82^. Encapsulations of such donors and acceptors inside organized assemblies or linked systems have been reported to regulate the diffusion dynamics of the radicals and can prolong their spin correlations along with an optimal separation between the two^49,83^. Figure 5 shows the effect of MF on the transient absorption spectra of MQ-ADN, ^Au^MQ-ADN, and ^Fe^MQ-ADN in the SDS micelles. It is found that the transient absorption increases with increasing MF from 0 to 0.08 T, especially in the region where exists the signature of RIPs, i.e., MQH^·^ and ADN(−H)^·^ and ADN^·+^ and MQ^·−^, generated from HAT and ET respectively as the prime mechanisms for the individual system. The increment of the triplet absorption spectra with MF over the signature region of the radicals is persistent over an extended range of time window often beyond 8 μs, indicating that the spin correlation in these RPs is long. We observe a relatively higher field effect for AuNP compared to FeNP. The higher MFE can be ascribed to optically induced magnetization of the plasmonic AuNP, compared to a demagnetization in FeNP, as reported in time-resolved Faraday rotation measurements^84,85^. Particularly noteworthy to mention are the stable lifetimes of the photogenerated RPs in the presence of the NPs. We anticipate that electron hopping from one donor ADN to nearby adsorbed ADN in the presence of FeNP, can prolong the lifetime of the spin-correlated RPs. Moreover, the superparamagnetic nano-surfaces of FeNP, can maintain the spin correlations after electron hopping to a second donor, particularly in the presence of MF; hence we observe such long lifetimes for FeNPs. Several reports of electron hopping between adjacent donors and elongation of the spin decoherence time for the entangled spin correlated radicals in the bridged donor–acceptor system have been reported^86–89^.

**Figure 6:**
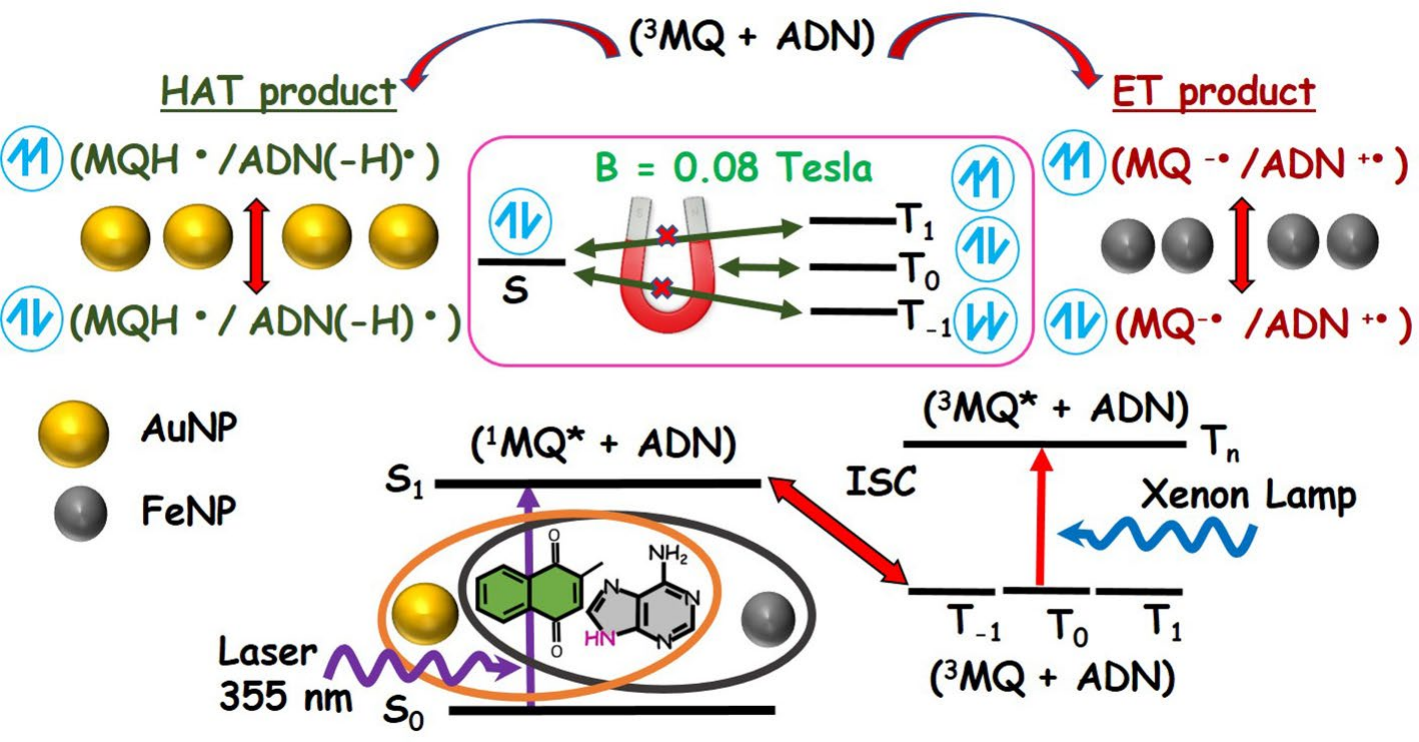
Plausible reaction pathways showing the various excited state photophysical and photochemical processes (ISC, ET and HAT) in the presence of 0.08 T magnetic field. The box in the middle shows the splitting of the non-degenerate triplet energy levels in presence of the field. The orange ellipsoid indicates the reaction scheme in presence of AuNP, while the dark one reflects it for the FeNP.

The τ values obtained by biexponential fitting of the decay profiles of the transients at 380 nm, 420 nm, and 520 nm for the individual system are listed in Table 1. The presence of an external MF increases the lifetime of the radicals and radical ions, which are the components of spin correlated geminate RPs and RIPs, respectively. The faster component is due to RP decay in the micellar cage, whereas the slower one is due to the contribution from escaped radicals. It is evident that a decrease in the rate constant as well as an increase in a lifetime (τ) of RP/RIP is observed in the presence of the MF. The relative escape yields of the transients are obtained from Eqs. 1 and 2 (Table 2). It is found that with MF, the lifetime and correspondingly escape yield of the transients increases. This implies that the RPs/RIPs are generated initially with the triplet spin correlations of the free electrons generated from the interaction of ^3^MQ with others. In the presence of the external MF, the inter-conversion between triplet and corresponding singlet RP/RIP evolved through hyperfine interactions present in the system is retarded, and consequently, the lifetime, as well as escape yield of corresponding radical/radical ion, is increased (Fig. 6). The above results indicate the HAT predominates over the ET between MQ and ADN in the presence of AuNP, and the reverse happens with FeNP and both the phenomena are confirmed by MFE, which shows an increase in the lifetime as well as in the relative escape yields of the radical products. With AuNP the decay rate of MQH^·^ at 420 nm becomes 1.85 times slower than that in the absence of MF with enhancing the relative yield to 1.35. This confirms that the transient radicals are generated through HAT. Similarly, very little change is observed in absorbance and as well as in decay profile around 520 nm which indicates that the extent of ET is too small to be identified by MF. The transients which are formed either through HAT or ET, are further confirmed from time-resolved transient absorption spectra over the time range between 0.5 and 4.9 μs in the presence of MF shown in Fig. 4d–f. Compared to the counterpart Fig. 4a–c in the absence of MF, the radical products show (Fig. 4e, f) more distinct and stable features, due to enhancement in the lifetimes of the formed radicals in the presence of MF. The radical features for MQH^·^ at 420 nm for AuNP and MQ^·−^ at 520 nm for FeNP reflect hardly any drop in their transient absorption even after a delay time of 4.9 μs (Fig. 4b, c, e, f), indicating greater stability with MF.

**Table 2:** Radical escape yield (REY) at 1 μs and 4 μs time delay and Lifetime Yield (LY) at 380, 420, and 520 nm, respectively.

	Peak at 380 nm				Peak at 420 nm				Peak at 520 nm			
	REY		LY (%)		REY		LY ( %)		REY		LY (%)	
	1 μs	4 μs			1 μs	4 μs			1 μs	4 μs		
MQ	1.4	1.5	61	0	1.21	2.82	37	0	1.6	0.48	92	0
MQ-ADN	1.05	0.93	18	2	1.21	1.21	16	-	1.12	1.18	71	
^Au^MQ-ADN	1.38	1.14	16	0	1.35	1.33	70	33	1.1	1.25	-	-
^Fe^MQ-ADN	1.08	0.73	50	18	1.12	1.03	43	0	1.36	1.16	02	32

The confirmation of RIP formation in the case of FeNP is obtained by the growth of the decay curve around 380 nm as well as at 410 nm as shown in Figure S2 and S3. It is mentioned previously that the growth profile in respective wavelengths followed by slow decay indicates the formation of stable species with a longer lifetime of 3.09 μs as given in Table 2. However, the change of lifetime around 380 nm is negligible as it is the wavelength where the commencement of the formation of the species could be identified, hence MFE is negligible. Although we have appreciable absorbance around the growth profile at 410 nm, however the change in a lifetime, as well as relative escape yield, is too small to be evaluated. However, the lifetime of the transient around 520 nm is increased from 2.11 to 2.78 μs by application of MF with the increase in relative escape yield from 1.0 to 1.36 which confirms the presence of the radical ion MQ^·−^ generated through ET in ^Fe^MQ-ADN (Figure S4).

## Conclusions

In this report, we have revealed the underlying charge transfer kinetics of MQ with purine base ADN and their alterations in the presence of AuNP and FeNP. Due to preferential interactions of the NPs to MQ and ADN, a regulated switch over in the reaction kinetics between ET and HAT is identified. Introducing AuNP facilitates HAT, resulting in the efficient production of HAT radicals, while in the presence of FeNP, ET radical yields are dominated due to their preferential binding to ADN and MQ, respectively. We believe the utter confirmation of such facile charge transfer with MF will open up new avenues for rational designing and engineering of NPs for precise measurement of their efficacy. Studies on these NPs with adequate exposure to different photoinduced redox pathways can undoubtedly prevent their detrimental effects on the many existing natural catalytic reactions inside the cell. Any changes in these regular catalytic charge transfer pathways may result in the formation of unwanted ET and HAT radical intermediates exerting to severe toxic effects inside the cell. Counter-arguments of enhancement in the catalytic rate have also been reported with the nanoparticles; however, any adverse side effect on the naturally occurring reaction pathway also should be dealt carefully. The recent upsurge in the usages of AuNP and FeNP for drug delivery, diagnostic, sensing, imaging, and biomedical application, may come with an uphill task of perturbing other reaction pathways occurring simultaneously, and hence demand a careful design of metal nanoparticle-based biomedical products with minimal exposure to conventional biological processes. We believe in a more holistic approach for usages of such nanoparticle for biomedical based applications, including their long-term toxicity effects and any parallel side effects resulting from their photo physics.

## Supplementary information

Supplementary information.

## References

[1] Linic S, Christopher P, Xin H, Marimuthu A (2013). Catalytic and photocatalytic transformations on metal nanoparticles with targeted geometric and plasmonic properties. Acc. Chem. Res..

[2] Kelly KL, Coronado E, Zhao LL, Schatz GC (2003). The optical properties of metal nanoparticles: The influence of size, shape, and dielectric environment. J. Phys. Chem. B.

[3] Wang Y, Cai R, Chen C (2019). The nano-bio interactions of nanomedicines: Understanding the biochemical driving forces and redox reactions. Acc. Chem. Res..

[4] Yang X (2017). Plasmon–exciton coupling of monolayer MoS_2_–Ag nanoparticles hybrids for surface catalytic reaction. Mater. Today Energy.

[5] Lin W, Ren X, Cui L, Zong H, Sun M (2018). Electro-optical tuning of plasmon-driven double reduction interface catalysis. Appl. Mater. Today.

[6] Lin W (2017). Physical mechanism on exciton–plasmon coupling revealed by femtosecond pump-probe transient absorption spectroscopy. Mater. Today Phys..

[7] Chen G, Roy I, Yang C, Prasad PN (2016). Nanochemistry and nanomedicine for nanoparticle-based diagnostics and therapy. Chem. Rev..

[8] Lane LA, Qian X, Nie S (2015). SERS nanoparticles in medicine: From label-free detection to spectroscopic tagging. Chem. Rev..

[9] Lee N (2015). Iron oxide based nanoparticles for multimodal imaging and magnetoresponsive therapy. Chem. Rev..

[10] Suk JS, Xu Q, Kim N, Hanes J, Ensign LM (2016). PEGylation as a strategy for improving nanoparticle-based drug and gene delivery. Adv. Drug Deliv. Rev..

[11] Jokerst JV, Lobovkina T, Zare RN, Gambhir SS (2011). Nanoparticle PEGylation for imaging and therapy. Nanomedicine.

[12] Fuhrer R, Athanassiou EK, Luechinger NA, Stark WJ (2009). Crosslinking metal nanoparticles into the polymer backbone of hydrogels enables preparation of soft, magnetic field-driven actuators with muscle-like flexibility. Small.

[13] Lu G (2012). Imparting functionality to a metal–organic framework material by controlled nanoparticle encapsulation. Nat. Chem..

[14] Zhou L, He B, Huang J (2013). One-step synthesis of robust amine- and vinyl-capped magnetic iron oxide nanoparticles for polymer grafting, dye adsorption, and catalysis. ACS Appl. Mater. Interfaces.

[15] Park J-W, Shumaker-Parry JS (2014). Structural study of citrate layers on gold nanoparticles: Role of intermolecular interactions in stabilizing nanoparticles. J. Am. Chem. Soc..

[16] Al-Johani H (2017). The structure and binding mode of citrate in the stabilization of gold nanoparticles. Nat. Chem..

[17] Orel V (2015). Magnetic properties and antitumor effect of nanocomplexes of iron oxide and doxorubicin. Nanomed. Nanotechnol. Biol. Med..

[18] Hu Y, Mignani S, Majoral J-P, Shen M, Shi X (2018). Construction of iron oxide nanoparticle-based hybrid platforms for tumor imaging and therapy. Chem. Soc. Rev..

[19] Zhou W, Gao X, Liu D, Chen X (2015). Gold nanoparticles for in vitro diagnostics. Chem. Rev..

[20] Yang X, Yang M, Pang B, Vara M, Xia Y (2015). Gold nanomaterials at work in biomedicine. Chem. Rev..

[21] Dreaden EC, Alkilany AM, Huang X, Murphy CJ, El-Sayed MA (2012). The golden age: Gold nanoparticles for biomedicine. Chem. Soc. Rev..

[22] Samanta A, Zhou Y, Zou S, Yan H, Liu Y (2014). Fluorescence quenching of quantum dots by gold nanoparticles: A potential long range spectroscopic ruler. Nano Lett..

[23] Xue C, Xue Y, Dai L, Urbas A, Li Q (2013). Size- and shape-dependent fluorescence quenching of gold nanoparticles on perylene dye. Adv. Opt. Mater..

[24] Pacioni NL, González-Béjar M, Alarcón E, McGilvray KL, Scaiano JC (2010). Surface plasmons control the dynamics of excited triplet states in the presence of gold nanoparticles. J. Am. Chem. Soc..

[25] Zhao G-J, Han K-L (2012). Hydrogen bonding in the electronic excited state. Acc. Chem. Res..

[26] Zhao G-J, Liu J-Y, Zhou L-C, Han K-L (2007). Site-selective photoinduced electron transfer from alcoholic solvents to the chromophore facilitated by hydrogen bonding: A new fluorescence quenching mechanism. J. Phys. Chem. B.

[27] Sarangi MK (2013). Hydrogen bond sensitive probe 5-methoxy-1-keto-1,2,3,4-tetrahydro carbazole in the microheterogeneity of binary mixtures and reverse micelles. J. Phys. Chem. C.

[28] Harmatys KM, Overchuk M, Zheng G (2019). Rational design of photosynthesis-inspired nanomedicines. Acc. Chem. Res..

[29] Darcy J, Koronkiewicz B, Parada GA, Mayer JM (2018). A continuum of proton-coupled electron transfer reactivity. Acc. Chem. Res..

[30] Hammes-Schiffer S (2018). Controlling electrons and protons through theory: molecular electrocatalysts to nanoparticles. Acc. Chem. Res..

[31] Dogutan D, Nocera DG (2019). Artificial photosynthesis at efficiencies greatly exceeding that of natural photosynthesis. Acc. Chem. Res..

[32] Zhang Z, Zhang C, Zheng H, Xu H (2019). Plasmon-driven catalysis on molecules and nanomaterials. Acc. Chem. Res..

[33] Bolton JL, Trush MA, Penning TM, Dryhurst G, Monks TJ (2000). Role of quinones in toxicology. Chem. Res. Toxicol..

[34] Bolton JL, Dunlap T (2017). Formation and biological targets of quinones: Cytotoxic versus cytoprotective effects. Chem. Res. Toxicol..

[35] Schieber A (2018). Reactions of quinones—Mechanisms, structures, and prospects for food research. J. Agric. Food. Chem..

[36] Maresz K (2015). Proper calcium use: Vitamin K2 as a promoter of bone and cardiovascular health. Integr. Med. (Encinitas).

[37] Villa JKD, Diaz MAN, Pizziolo VR, Martino HSD (2017). Effect of vitamin K in bone metabolism and vascular calcification: A review of mechanisms of action and evidences. Crit. Rev. Food Sci. Nutr..

[38] Theuwissen E, Smit E, Vermeer C (2012). The role of vitamin K in soft-tissue calcification. Adv. Nutr..

[39] Vos M (2012). Vitamin K_2_ is a mitochondrial electron carrier that rescues Pink1 deficiency. Science.

[40] Jankowski, O. D., Hinman, A. W., Miller, G. M. & Inc, A. L. S. (2011).

[41] Dragh MA, Xu Z, Al-Allak ZS, Hong L (2017). Vitamin K2 prevents lymphoma in drosophila. Sci. Rep..

[42] Ivanova D (2018). Vitamin K: Redox-modulation, prevention of mitochondrial dysfunction and anticancer effect. Redox. Biol..

[43] Akiyoshi T, Matzno S, Sakai M, Okamura N, Matsuyama K (2009). The potential of vitamin K3 as an anticancer agent against breast cancer that acts via the mitochondria-related apoptotic pathway. Cancer Chemother. Pharmacol..

[44] Lamson DW, Plaza SM (2003). The anticancer effects of vitamin K. Altern. Med. Rev..

[45] Hitomi M (2005). Antitumor effects of vitamins K1, K2 and K3 on hepatocellular carcinoma in vitro and in vivo. Int. J. Oncol..

[46] Trachootham D, Alexandre J, Huang P (2009). Targeting cancer cells by ROS-mediated mechanisms: A radical therapeutic approach?. Nat. Rev. Drug Discov..

[47] Sengupta T, Sharmistha Dutta Choudhury A, Basu S (2004). Medium-dependent electron and H atom transfer between 2′-deoxyadenosine and menadione: A magnetic field effect study. Am. Chem. Soc..

[48] Hore PJ, Mouritsen H (2016). The radical-pair mechanism of magnetoreception. Annu. Rev. Biophys..

[49] Sarangi MK, Basu S (2011). Associated electron and proton transfer between Acridine and Triethylamine in AOT reverse micelles probed by laser flash photolysis with magnetic field. Chem. Phys. Lett..

[50] Sarangi MK, Basu S (2011). Photophysical behavior of acridine with amines within the micellar microenvironment of SDS: A time-resolved fluorescence and laser flash photolysis study. Phys. Chem. Chem. Phys..

[51] Kattnig DR (2016). Chemical amplification of magnetic field effects relevant to avian magnetoreception. Nat. Chem..

[52] Behera R (2019). Redox modifications of carbon dots shape their optoelectronics. J. Phys. Chem. C.

[53] Sau A, Bera K, Mondal P, Satpati B, Basu S (2016). Distance-dependent electron transfer in chemically engineered carbon dots. J. Phys. Chem. C.

[54] Cohen AE (2009). Nanomagnetic control of intersystem crossing. J. Phys. Chem. A.

[55] Chakraborty SD (2016). Development of a triplet–triplet absorption ruler: DNA- and chromatin-mediated drug molecule release from a nanosurface. J. Phys. Chem. B.

[56] Chakraborty S (2018). Low magnetic field induced surface enhanced transient spin-trajectory modulation of a prototype anticancer drug sanguinarine on a single domain superparamagnetic nanosurface. J. Phys. Chem. C.

[57] Sarangi MK, Dey D, Basu S (2011). Influence of heterogeneity of confined water on photophysical behavior of acridine with amines: A time-resolved fluorescence and laser flash photolysis study. J. Phys. Chem. A.

[58] Sarangi MK, Mitra A, Basu S (2012). Prototropic interactions of pyrimidine nucleic acid bases with acridine: A spectroscopic investigation. J. Phys. Chem. B.

[59] Lakowicz JR (2006). Principles of Fluorescence Spectroscopy.

[60] Sarangi MK, Bhattacharyya D, Basu S (2012). Influence of 2′-deoxy sugar moiety on excited-state protonation equilibrium of adenine and adenosine with acridine inside SDS micelles: A time-resolved study with quantum chemical calculations. ChemPhysChem.

[61] Bergeron F, Houde D, Hunting DJ, Wagner JR (2004). Electron transfer in DNA duplexes containing 2-methyl-1,4-naphthoquinone. Nucleic Acids Res..

[62] Hubig SM, Bockman TM, Kochi JK (1997). Identification of photoexcited singlet quinones and their ultrafast electron-transfer vs intersystem-crossing rates. J. Am. Chem. Soc..

[63] Fraser DD, Bolton JR (1994). Intramolecular photochemical electron transfer. 8. Decay of the triplet state in a porphyrin–quinone molecule. J. Phys. Chem..

[64] Wagner JR, Lier JEV, Johnston LJ (1990). Quinone sensitized electron transfer photooxidation of nucleic acids: Chemistry of thymine and thymidine radical cations in aqueous solution. Photochem. Photobiol..

[65] Amada I, Yamaji M, Sase M, Shizuka H (1995). Laser flash photolysis studies on hydrogen atom abstraction from phenol by triplet naphthoquinones in acetonitrile. J. Chem. Soc. Faraday Trans..

[66] Becker RS, Natarajan L (1993). Comprehensive absorption, photophysical/chemical, and theoretical study of 2-5-ring aromatic hydrocarbon diones. J. Phys. Chem..

[67] Pan Y (2006). Studies on photoinduced H-atom and electron transfer reactions of o-naphthoquinones by laser flash photolysis. J. Phys. Chem. A.

[68] Banerjee S, Dutta Choudhury S, Dasgupta S, Basu S (2008). Photoinduced electron transfer between hen egg white lysozyme and anticancer drug menadione. J. Lumin..

[69] Kang H, Lee KT, Jung B, Ko YJ, Kim SK (2002). intrinsic lifetimes of the excited state of DNA and RNA bases. J. Am. Chem. Soc..

[70] Middleton CT (2009). DNA excited-state dynamics: from single bases to the double helix. Annu. Rev. Phys. Chem..

[71] Barbatti M (2010). Relaxation mechanisms of UV-photoexcited DNA and RNA nucleobases. Proc. Natl. Acad. Sci. USA.

[72] Sharma S, Lee JK (2002). Acidity of adenine and adenine derivatives and biological implications. A computational and experimental gas-phase study. J. Org. Chem..

[73] Krishnamurthy R (2012). Role of pKa of nucleobases in the origins of chemical evolution. Acc. Chem. Res..

[74] Kimura-Suda H, Petrovykh DY, Tarlov MJ, Whitman LJ (2003). Base-dependent competitive adsorption of single-stranded DNA on gold. J. Am. Chem. Soc..

[75] Östblom M, Liedberg B, Demers LM, Mirkin CA (2005). On the structure and desorption dynamics of DNA bases adsorbed on gold: A temperature-programmed study. J. Phys. Chem. B.

[76] Kundu J (2009). Adenine− and adenosine monophosphate (AMP)−Gold binding interactions studied by surface-enhanced Raman and infrared spectroscopies. J. Phys. Chem. C.

[77] Carnerero JM, Sánchez-Coronilla A, Martín EI, Jimenez-Ruiz A, Prado-Gotor R (2017). Quantification of nucleobases/gold nanoparticles interactions: Energetics of the interactions through apparent binding constants determination. Phys. Chem. Chem. Phys..

[78] Chen Q, Frankel DJ, Richardson NV (2002). Self-assembly of adenine on Cu(110) surfaces. Langmuir.

[79] Zheng X (2011). catalytic gold nanoparticles for nanoplasmonic detection of DNA hybridization. Angew. Chem. Int. Ed..

[80] Vogt N, Dorofeeva OV, Sipachev VA, Rykov AN (2009). Molecular structure of 9H-adenine tautomer: Gas-phase electron diffraction and quantum-chemical studies. J. Phys. Chem. A.

[81] Raczyńska ED, Kolczyńska K, Stępniewski TM, Kamińska B (2013). On relation between prototropy and electron delocalization for neutral and redox adenine—DFT studies. Comput. Theor. Chem..

[82] Steiner UE, Ulrich T (1989). Magnetic field effects in chemical kinetics and related phenomena. Chem. Rev..

[83] Closs GL, Forbes MDE, Norris JR (1987). Spin-polarized electron paramagnetic resonance spectra of radical pairs in micelles: Observation of electron spin–spin interactions. J. Phys. Chem..

[84] Hsia C-H, Chen T-Y, Son DH (2008). Size-dependent ultrafast magnetization dynamics in iron oxide (Fe_3_O_4_) nanocrystals. Nano Lett..

[85] Cheng OH-C, Son DH, Sheldon M (2020). Light-induced magnetism in plasmonic gold nanoparticles. Nat. Photonics.

[86] Wu Y (2018). Covalent radical pairs as spin qubits: Influence of rapid electron motion between two equivalent sites on spin coherence. J. Am. Chem. Soc..

[87] Olshansky J, Krzyaniak MD, Young RM, Wasielewski MR (2019). photogenerated spin-entangled qubit (radical) pairs in DNA hairpins: Observation of spin delocalization and coherence. J. Am. Chem. Soc..

[88] Forbes MDE (2019). Beam me up scotty. Nat. Chem..

[89] Rugg BK (2019). Photodriven quantum teleportation of an electron spin state in a covalent donor–acceptor–radical system. Nat. Chem..

