# Supplementary material for: Metal nanoparticle alters adenine induced charge transfer kinetics of vitamin K3 in magnetic field
Source: Sci Rep. 2020 Oct 28;10:18454. doi: 10.1038/s41598-020-75262-8 (PMC7595215; doi:10.1038/s41598-020-75262-8)
Supplement: Supplementary file 1 — Supplementary information. [file 41598_2020_75262_MOESM1_ESM.pdf]

Supplementary Information

Metal Nanoparticle Alters Adenine Induced Charge Transfer Kinetics of Vitamin K3 in Magnetic Field

Ranjan Kumar Behera[#], AbhishekSau[†$], Leepsa Mishra[#], Sankalan Mondal[#], Kallol Bera[†&], Satish Kumar[#], Samita Basu [†], Manas Kumar Sarangi*[, #]

[#]Department of Physics, Indian Institute of Technology Patna, India

[†]Chemical Sciences Division, Saha Institute of Nuclear Physics Kolkata, India

[$]Department of Molecular and Cellular Medicine, Texas A&M University, USA,

[&]Division of Biology & Biological Engg, California Institute of Technology, Pasadena, USA

Corresponding Author
* mksarangi@iitp.ac.in

Supplementary Figures for Materials and Methods:

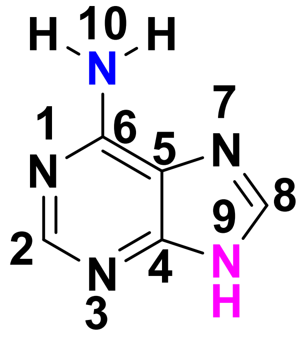

Supplementary Scheme-1 Adenine numbering showing the possible acidic sites (N9 and N10)

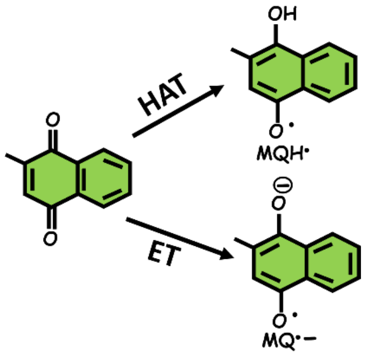

Supplementary Scheme-2 Probable structure of $MQH^{\cdot}$ & $MQ^{\cdot -}$

Supplementary Figures for Results and Discussions:

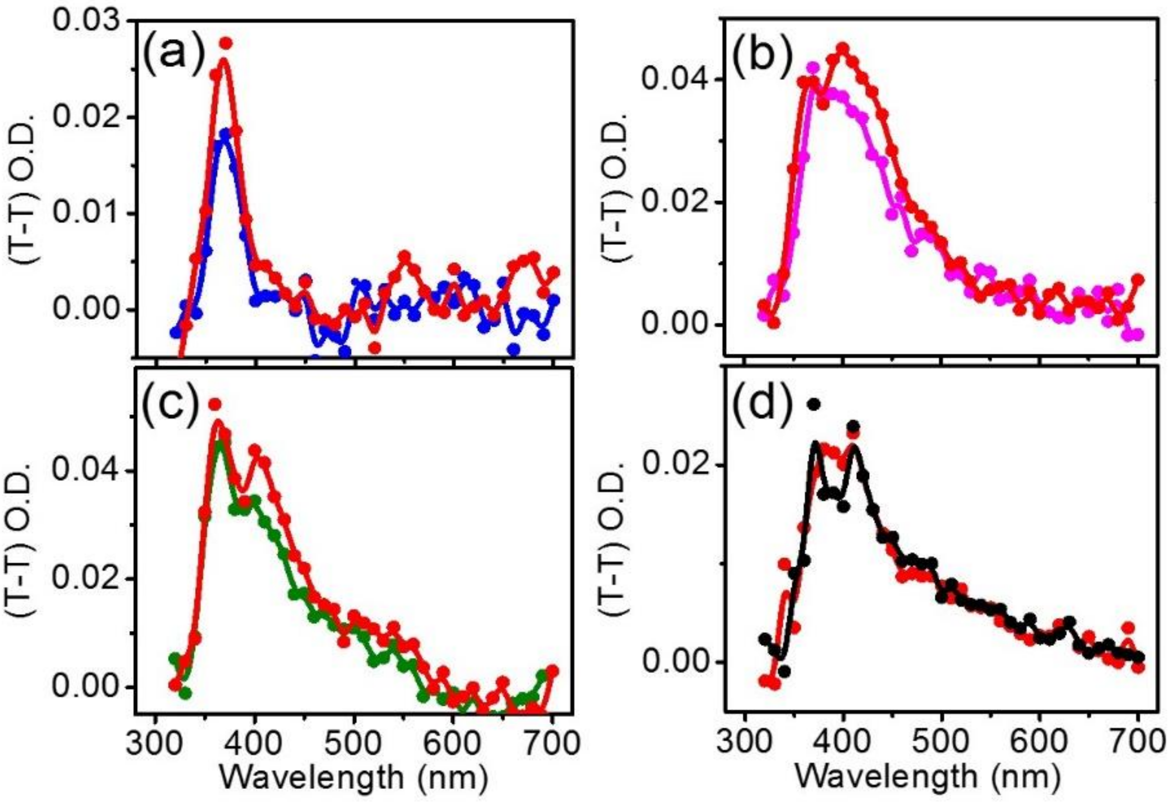

Figure S1: (a,b,c,d) are transient absorption spectra between pump and probe pulse at 4μs delay of MQ (blue), MQ-ADN (magenta), ^{Au}MQ-ADN (olive) and, ^{Fe}MQ-ADN (black) respectively and all of the red spectra represent the presence of magnetic field of their corresponding spectra.

.

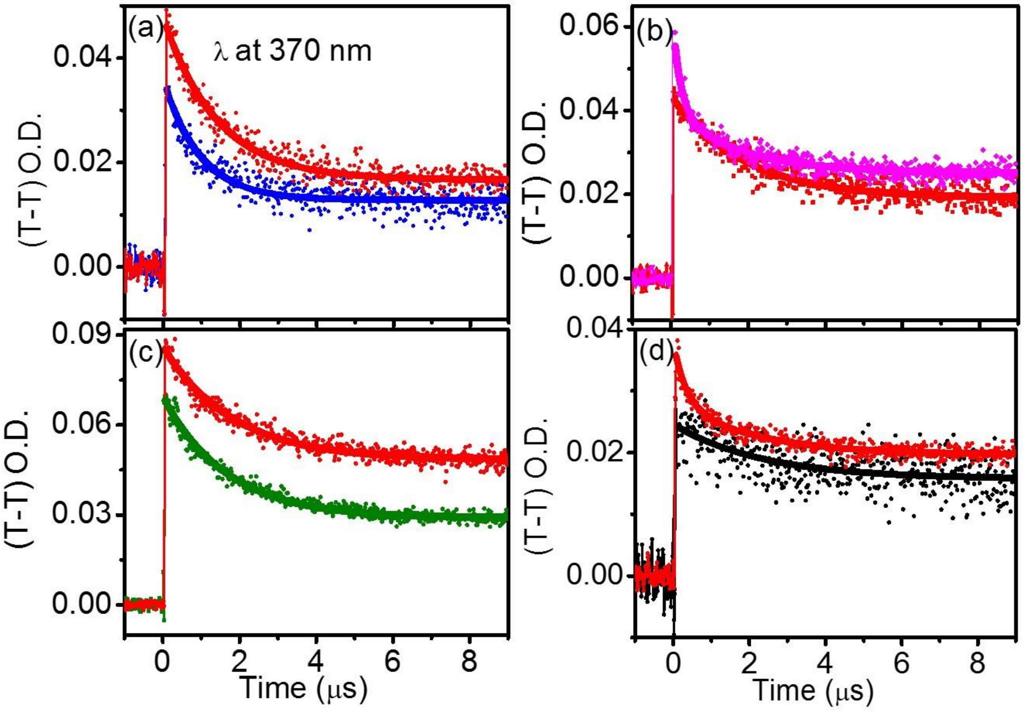

Figure S2: a, b, c, d shows the decay profiles at 370 nm wavelength generated by laser flash photolysis in the absence and presence (red all) of magnetic field of MQ (blue), MQ-ADN (magenta), ^{Au}MQ-ADN (olive) and, ^{Fe}MQ-ADN (black) respectively.

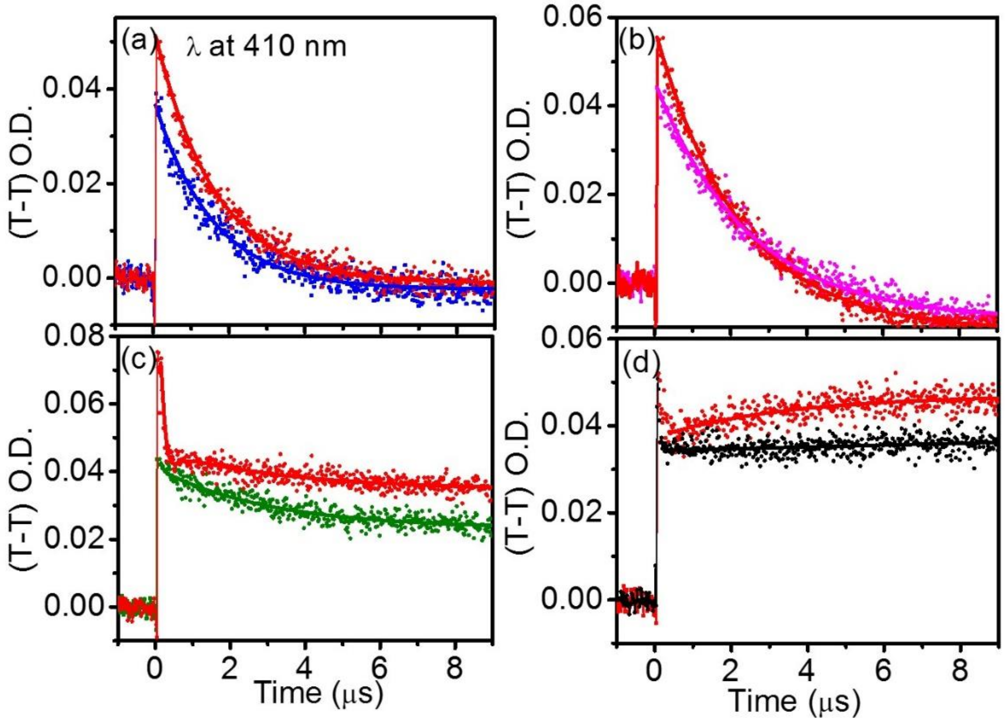

Figure S3: a, b, c, d shows the decay profiles at 410 nm wavelength generated by laser flash photolysis in the absence and presence (red all) of magnetic field of MQ (blue), MQ- ADN (magenta), ^{Au}MQ-ADN (olive) and, ^{Fe}MQ-ADN (black) respectively.

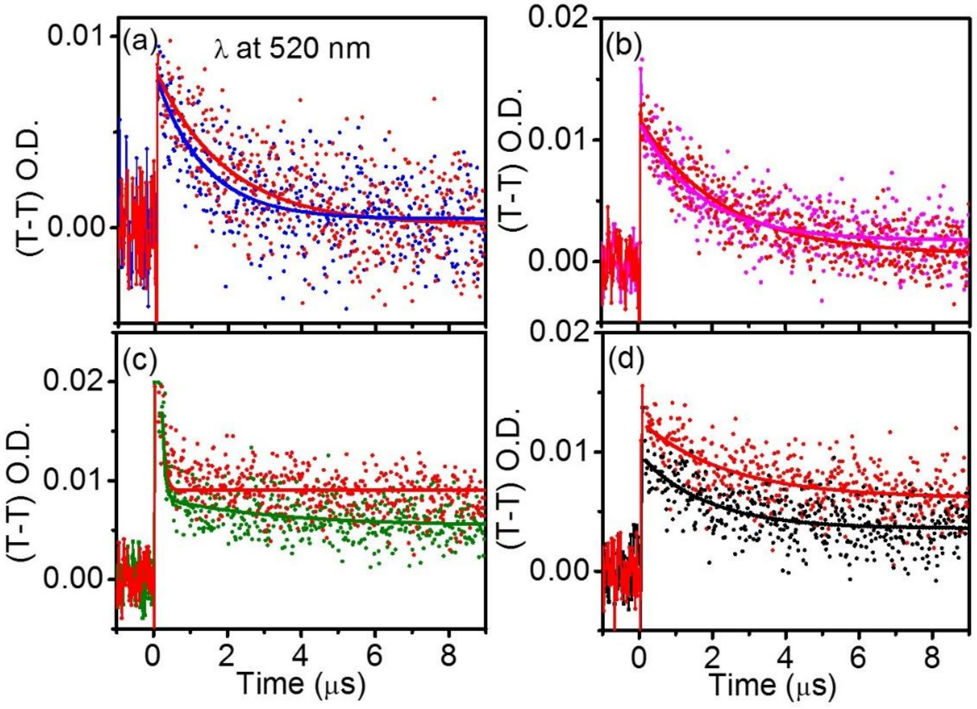

Figure S4 : a, b, c, d shows the decay profiles at 510 nm wavelength generated by laser flash photolysis in the absence and presence (red all) of magnetic field of MQ (blue), MQD (magenta), ^{Au}MQ-ADN (olive) and, ^{Fe}MQ-ADN (black) respectively.

	^{370}Area (%)	^{410}Area (%)	^{520}Area (%)	410@ 370 (%)	520@ 370 (%)	370 @ 410 (%)	520@ 410(%)	370 @ 520(%)	410@ 520 (%)
MQ	14.7	57.5	27.8	43.9	2.8	0.9	7.8	0	5.8
MQ-ADN	24.8	48.8	26.4	36.1	1.7	12.1	6	0	2.5
^{Au}MQ-ADN	24.8	65.1	10.1	41.2	0.1	6.4	1	0	17.9
^{Fe}MQ-ADN	13.6	50.3	36.1	41.5	0.2	0.5	2.8	0	1.8

Table ST1: the calculation of areal contribution from the deconvolution curve of MQ (blue), MQ-ADN (magenta), ^{Au}MQ-ADN (olive) and, ^{Fe}MQ-ADN (black) respectively shown in Figure-3.
